# The Uncharted Landscape of Rare Endocrine Immune-Related Adverse Events

**DOI:** 10.3390/cancers15072016

**Published:** 2023-03-28

**Authors:** Chrysoula Mytareli, Dimitrios C. Ziogas, Athina Karampela, Petros Papalexis, Vasiliki Siampanopoulou, Alexandros Lafioniatis, Olga Benopoulou, Helen Gogas, Anna Angelousi

**Affiliations:** 1 First Department of Internal Medicine, Unit of Endocrinology, Laikon General Hospital, National and Kapodistrian University of Athens, 11527 Athens, Greece; 2 First Department of Internal Medicine, Unit of Medical Oncology, Laikon General Hospital, National and Kapodistrian University of Athens, 11527 Athens, Greece; 3 First Department of Internal Medicine, Laikon General Hospital, National and Kapodistrian University of Athens, 11527 Athens, Greece

**Keywords:** immune checkpoint inhibitors, rare adverse events, endocrinopathies, autoimmunity, anti-PD-1, anti-PD-L1, anti-CTLA-4

## Abstract

**Simple Summary:**

Immune checkpoint inhibitors (ICIs) are considered to be the standard of care in multiple types of cancers. However, ICIs are implicated in a wide range of side effects, referred to as immune-related adverse events (irAEs). Endocrine irAEs, especially thyroid dysfunction and hypophysitis, are some of the most frequently reported side effects. However, several other endocrine irAEs have been described less frequently and can be encountered more recurrently as the use of ICIs expands. This systematic review includes all published cases with rare and very rare endocrine irAEs, emphasizing mostly their diagnostic and therapeutic approach as well as their underlying pathogenesis. The aim of our study is to raise clinical awareness related to early diagnosis and proper treatment of these rare but, in some cases, potentially fatal endrocine irAEs.

**Abstract:**

Immune checkpoint inhibitors (ICIs) have been approved for the treatment of many cancers, either in adjuvant or metastatic settings. Regarding safety, endocrine adverse events (AEs) are some of the most common AEs in ICI-treated patients, with thyroid dysfunction and hypophysitis being the most frequent disorders. However, there are also some rare and very rare immune-related (ir) endocrine complications (incidence between ≥1/10,000 to <1/1000 and <1/10,000, respectively, according to the established classification) that have been reported in isolated case reports, with limited data about their management. In this systematic review, we summarize all published cases with primary adrenal insufficiency, central diabetes insipidus, primary hypoparathyroidism, lipodystrophy, osteoporosis, hypergonadotrophic hypogonadism, or Cushing disease and discuss their diagnostic and therapeutic approaches as well as the current knowledge on their pathophysiology. In these ICI-treated cancer patients, the presentation of symptoms unrelated to their underlying malignancy has led to further diagnostic tests, including hormonal profile and functional assays which subsequently confirmed endocrinopathy, while the assessment of autoantibodies was rarely available. In most of these cases, the exact pathogenesis remained unknown, and the endocrine dysfunction was permanent, requiring lifelong supplementation. Although endrocine irAEs are rare, physicians must be aware of these irAEs to recognize them on time and treat them appropriately.

## 1. Introduction

The introduction of immune checkpoint inhibitors (ICIs) (anti-CTLA-4, anti-PD-1 inhibitors, and anti-PD-L1 inhibitors) in adjuvant and metastatic settings has revolutionized the therapeutic approach of several cancer types [1]. Immune checkpoints are small molecules on the cell surface of T lymphocytes which play vital roles in maintaining immune homeostasis and self-tolerance and in modulating the duration and amplitude of immune response against tumor cells [1]. Several monoclonal antibodies target and block inhibitory checkpoints allowing adaptive immunity to attack tumor cells [1]. However, this amplification of the immune response induced by ICIs can also be potentially directed against healthy human tissues and cause a wide range of side effects, with variable degrees of severity, known as immune-related adverse events (irAEs) that include dermatologic, gastrointestinal, respiratory, hepatic, endocrine, and other less common inflammatory toxicities. Four mechanisms have been proposed to explain the onset of irAEs [2]. The first mechanism includes increased T cell activity against antigens that are present both in tumors and in healthy cells. An increase in pre-existing autoantibodies and the expansion of T cell populations capable of releasing proinflammatory cytokines after ICI administration are two other suggested pathogenetic mechanisms, while the last theory suggests that checkpoint molecules (CTLA-4, PD-1, and PD-L1) can be found not only on T lymphocytes but also on other normal cells. Direct binding of ICIs with tissue expressing immune checkpoints may trigger cytotoxic immune reactions that eventually lead to organ-specific toxicity.

IrAEs are evaluated and treated using the Common Terminology Criteria for Adverse Events, the European Society for Medical Oncology guideline, and the American Society of Clinical Oncology guideline [3]. In general, discontinuation of ICI therapy is recommended for grade ≥2–3 irAE toxicities [3]. It is noteworthy that the management of immune-related endocrinopathies is quite different from other irAEs since high-dose corticosteroids are rarely required for the treatment of endocrine complications and, in addition, most endocrine dysfunctions are frequently irreversible regardless of ICI cessation [3]. Thus, the development of endocrinopathies does not necessarily prompt the interruption of ICI treatment [4]. According to the recent European Society of Endocrinology (ESE) clinical practice guideline [4], thyroid dysfunction represents the most common endocrine irAEs. The overall incidence of ICI-induced hypophysitis ranges between 1.8 and 17% [4,5] and ICI-induced diabetes mellitus (DM) between 0.9 and 2% [6].

In addition to the aforementioned relatively frequent endocrine irAEs, several other endocrine disorders have also been reported in the literature whose incidences are difficult to estimate because of their rarity. Herein, we review the existing evidence and collect all currently published cancer cases that underwent immunotherapy and developed these poorly described, yet exceedingly interesting, endocrine irAEs, including primary adrenal insufficiency, central diabetes insipidus, primary hypoparathyroidism, hypergonadotrophic hypogonadism, lipodystrophy, osteoporosis, or Cushing disease. Regardless of the underlying malignancy and the type of administered ICI, the entire diagnostic and therapeutic management is also thoroughly discussed.

## 2. Materials and Methods

This systematic review was carried out according to the Preferred Reporting Items for Systematic Reviews and Meta-analyses (PRISM), http://www.prisma-statement.org, accessed on the 14 October 2022. This study is registered with PROSPERO, number CRD42022367385.

### 2.1. Data Sources and Search Strategy

**Figure 1:**
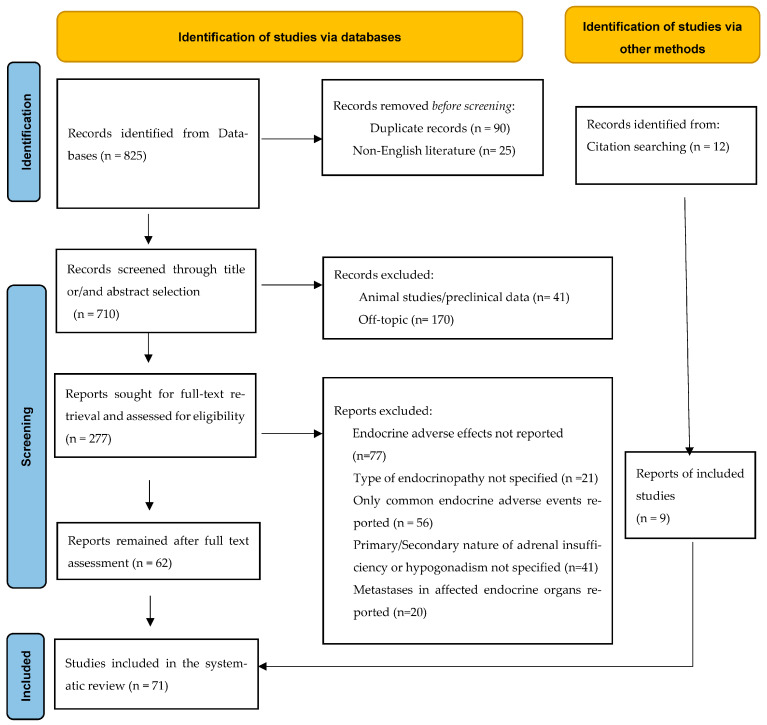
Flow diagram of the literature search strategy, according to the Preferred Reporting Items for Systematic Reviews and Meta-analyses (PRISMA) 2020 guidelines.

The databases PubMed, EMBASE, EMCARE, Web of Science, and COCHRANE Library were searched up to 28 October 2022 to identify potentially relevant studies. After the initial title and abstract screening, we assessed the full texts of studies to be potentially included and hand-searched their reference lists to gather additional relevant publications. The search terms included the following: “primary adrenal insufficiency”, “adrenalitis”, “Addison’s disease”, “diabetes insipidus”, “panhypopituitarism”, “hypoparathyroidism” “hypocalcemia”, “primary hypogonadism”, “orchitis”, “hypergonadotropic hypogonadism”, “infertility”, “spermatogenesis”, “osteoporosis”, “lipodystrophia”, “endocrine complications”, “endocrinopathies”, “immune checkpoint inhibitors”, “PD-1 inhibitor”, “PD-L1 inhibitor”, “CTLA-4 inhibitor”, “pembrolizumab”, “nivolumab”, “ipilimumab”, “atezolizumab”, “cemiplimab”, “avelumab”,”dostarlimab”, “tremelimumab”, “durvalumab”. The above keywords were combined with the Boolean operators AND and OR. The literature search was conducted by two independent investigators (C.M. and A.A.) and the selection of retrieved reports, including the numbers of the records identified or excluded and the reasons for exclusions, are represented in Figure 1. According to the PRISMA 2020 statement, our flow diagram depicts the flow of information through the different phases of the screening process, mapping out the number of records identified, included, and excluded as well as the reasons for exclusions [7]. All available data on clinical and diagnostic features, management, and outcomes of these cases were collected. Data extraction was undertaken by two investigators (A.K. and P.P.) independently. The level of initial agreement was assessed using Kappa statistics. Any discrepancy was resolved by a third independent investigator (D.C.Z.), who reviewed the data extraction.

### 2.2. Eligibility Criteria for Articles of Inclusion

According to the Food and Drug Administration (FDA) and the European Commission, adverse drug reactions are classified into 5 types: very common (≥1/10), common (from ≥1/100 to <1/10), uncommon (from ≥1/1000 to <1/100), rare (from ≥1/10,000 to <1/1000), and very rare (<1/10,000) [8]. Thus, eligible studies were considered to be all studies (e.g., case reports, case series, cohort studies, etc.) that included cancer patients (regardless of their type of malignancy) who were treated with an ICI-based regimen (as a single agent or in combinatorial regimens with other ICI or chemotherapy) and who experienced rare and very rare endocrine irAEs. 

## 3. Literature Review Results

A total of 825 articles were initially retrieved from the search of the databases. After excluding duplicates (*n* = 90) and non-English literature (*n* = 25), 710 articles remained. Two of the authors independently examined all potentially eligible titles and abstracts to identify articles of interest, from which 433 articles were excluded because they did not include primary or clinical or relevant data. Based on the full text assessment of the remaining 277 studies, *n* = 77 were excluded because they reported other than endocrine irAEs and *n* = 21 studies were also excluded because they presented a total rate of immunotherapy-induced endocrinopathies without specifying the type of endocrinopathy. Fifty-six studies were further excluded because they reported only common endocrine complications, *n* = 41 were excluded because the origin of the described endocrine disorders was not specified (primary vs. secondary adrenal insufficiency or primary vs. secondary hypogonadism), and the last *n* = 20 studies were also excluded because of the detection of metastases in the affected endocrine organ. Nine additional reports were also identified and included in our analysis through hand-searched reference lists. Finally, a total of 71 studies including case reports (*n* = 56), case series (*n* = 5), cohort studies (*n* = 3), one cross-sectional study, one phase I clinical study, and pharmacovigilance retrospective studies (*n* = 5), were included in our study (Figure 1). 

### 3.1. Primary Adrenal Insufficiency (PAI)

#### 3.1.1. Background

Adrenal insufficiency (AI) induced by ICI is prevalently secondary (SAI), resulting from either an anterior hypophysitis or selective damage of ACTH-producing cells in the pituitary [9]. In a recent systematic review, the incidence of PAI was reported as 5.3% among all ICI-induced AI events (*n* = 206), whereas the majority of cases (92.7%) was attributed to SAI and the remaining cases (1.9%) were attributed to mixed-type AI [9]. An estimation of the precise incidence of ICI-induced PAI is difficult, because in the majority of the studies there is insufficient data regarding the distinction between PAI and SAI which can often lead to a misdiagnosis, and finally an underestimation of the PAI cases. 

#### 3.1.2. Case Studies 

There are three pharmacovigilance retrospective studies reporting data on ICI-induced PAI cases. The first study by [10] reported an incidence of ICI-induced PAI of 0.9% (451 PAI cases (45 definite, and 406 possible) out of 50.108 cases with irAEs) according to the WHO’s VigiBase from September 2008 to October 2018. The majority of patients were treated with ICI monotherapy: 58.5% were on anti–PD-1 or anti–PD-L1 treatment and 23.6% on anti–CTLA-4 treatment. Only 18% of patients with ICI-associated PAI had received combination ICI therapy. The median time to onset of symptoms since the initiation of immunotherapy was 120 days (range, 6–576) based on the data of 120 out of 451 patients. ICI-related PAI was associated with significant morbidity (≥90% severe) and mortality (7.3%). The second study by [11] reported an incidence of ICI-induced PAI of 0.86% (1180 PAI cases out of 137.566 cases with irAEs) based on the FDA Adverse Event Reporting System (FAERS) database from 2007 to 2020. The incidence of PAI was 0.77% (578/74,605) in patients on PD-1 inhibitors, 0.68% (160/23,445) in patients on PD-L1 inhibitors, and 0.6% (97/16,071) in patients on CTLA-4 inhibitors. In patients treated with combination therapy of ipilimumab and nivolumab the incidence was 1.47% (345/23,445). Patients on PD-1 inhibitors had a significantly higher risk of PAI compared to PD-L1 inhibitors (χ^2^ = 5.14, *p* = 0.022) but a lower risk of PAI compared to the combination therapy group (χ^2^ = 92.88, *p* < 0.001). In terms of prognosis, 937 cases (79.4%) had severe PAI compared to 243 cases (20.6%) in which PAI was considered to be mild. Among the severe cases, 53.5% of the cases necessitated hospitalization, 14% were life-threatening cases, and 11.9% of the cases led to death. The last study by [12] reported 11 cases (0.002%) of adrenal complications out of 534.688 cases with irAEs based on the Japanese Adverse Drug Event Report database from April 2004 to June 2018. 

Twenty-seven studies with detailed patients’ features have been published from 2013 to 2022 and are included in Table 1 [13,14,15,16,17,18,19,20,21,22,23,24,25,26,27,28,29,30,31,32,33,34,35,36,37,38,39]. These studies describe a total of 29 patients who experienced PAI following ICI therapy for various types of malignancy. Patients’ ages ranged from 14 to 79 years (mean ± SD: 58.9 ± 14.4 years). Three patients (10.3%) were treated with anti-CTLA-4 monotherapy, 20 patients (69%) were treated with anti-PD-1/L1 monotherapy, and the remaining six patients (20.7%) were treated with anti-CTLA-4 and anti-PD-1 combination therapy. In at least eight cases [16,21,23,24,27,34,37], ICIs were chosen as the first-line systematic therapy for advanced disease, whereas, in four cases ICI, treatment was combined with a tyrosine kinase inhibitor [13,22,27,29], which has also been implicated for PAI development [40]. The ICI-induced PAI was diagnosed from 10 days to 1 year post ICI initiation. In four cases, PAI was also associated with other autoimmune endocrinopathies such as DM and thyroiditis, leading to a diagnosis of autoimmune polyendocrine syndrome type 2 (APS-2) [13,16,17,28]. The majority of these reported cases (20/27, 74.1%) presented with severe or life-threatening symptoms (grade III/IV). Follow-up data were available for only nine patients from whom the vast majority (7/9, 77.8%) presented permanent PAI requiring lifelong steroid substitution, whereas only two cases experienced remission of their PAI confirmed with physiological response of cortisol in the synacthen test [26,34]. 

The assessment of antibodies was inconsistently reported throughout the studies. Six cases out of 10 [13,17,23,26,28,39] presented positive 21-hydroxylase antibodies (21-OHAbs), while antibodies were negative in four cases [20,29,35]. The 21-OHAbs and adrenal cortex antibodies (ACA) were both positive in two cases [13,23]. In the rest of the cases with confirmed cortisol deficiency, the diagnosis of PAI was established mainly based on elevated ACTH or plasma renin activity or concentration in combination with low serum aldosterone concentration and electrolyte disturbances. It is worthwhile noting that, in a few cases [16,27,28,30,36,37], the authors concluded a PAI diagnosis without providing data of the “classical” tests which are mandatory for the establishment of PAI.

Adrenal imaging was performed in 20 cases, through *computed tomography* (CT) (*n* = 12) or *fluorodeoxyglucose* positron emission tomography-CT (FDG-PET/CT) (*n*= 4), ultrasound (*n* = 1), or CT or MRI (*n* = 3), and was normal in 14 (70%) cases. Three patients presented adrenal cortical atrophy [13,21,39], one patient presented bilateral adrenal enlargement [25], and two patients [24,30,38] presented symmetrically and smoothly enlarged adrenal glands with increased metabolic activity in PET-FDG suggesting adrenal inflammation. 

Finally, a recent population-based cohort study that included 418 patients with melanoma treated with ICIs reported an unusual high incidence (8%) of PAI [41]. However, these findings should be interpreted with caution since no data were provided on the used diagnostic approach.

#### 3.1.3. Pathophysiology

The pathophysiology of ICI-related PAI remains unknown but is likely mediated by autoimmune activation caused by ICI. Cytotoxic T lymphocytes are considered to be the main cellular mediators of the adrenal gland destruction, while 21-OHAbs in the serum, although not directly involved in the pathogenesis of autoimmune forms of PAI [42], remain reliable disease biomarkers [43]. In addition, adrenal cortex antibodies (ACA) have also been identified in patients with autoimmune PAI, including steroid 17-α-hydroxylase and the cholesterol side-chain cleavage enzyme [43]. Over the course of autoimmune PAI, the three layers of the cortex are progressively destroyed and replaced by fibrous tissue. Despite continuous loss of adrenocortical cells, the disease may underlie subclinically for a long period of time and may not manifest itself until 90% of the cells are destroyed [42].

In the case of ICI-induced PAI, adrenal inflammation has also been confirmed by FDG-PET scan in which both glands appear hypermetabolic. Indeed, there is evidence of both humoral and cell-mediated immune mechanisms directed at the adrenal cortex. The rapid time to occurrence of PAI (a few days in some cases) may reflect a cytotoxic T cell-mediated destruction of the adrenal cortex [10]. Antibodies directed against steroidogenic enzymes have also been reported in cases of ICI-induced PAI, as described above.

Certain genotypes have been associated with an increased risk for developing PAI as an isolated disorder or as part of APS-2 [42,44]. These PAI-related genes encode immunological proteins such as CTLA-4 and human leukocyte antigen (HLA) [42]. Interestingly, in two cases with ICI-related APS-2, HLA typing was performed and revealed DRB1*04 and DQB1*03 high-risk haplotypes [16,17]. In these patients with genetic susceptibility, ICIs could potentially trigger the clinical onset of APS-2.

#### 3.1.4. Clinical Presentation

PAI may manifest as either an acute or chronic condition. In particular, the chronic form of PAI may be overlooked or confused with cancer-related symptoms, as symptoms are rather nonspecific and include weakness, anorexia, musculoskeletal pain, weight loss, abdominal pain, nausea, and vomiting. A specific sign of chronic PAI is hyperpigmentation that predominantly affects areas of the skin subjected to pressure. The onset of PAI is often gradual and may go undetected until an illness or other stress precipitates an adrenal crisis. An adrenal crisis is a life-threatening condition. Clinical features include vomiting, abdominal pain, severe hypotension, or hypovolemic shock associated with hyponatremia, hyperkalemia, or hypoglycemia [45].

#### 3.1.5. Diagnosis

According to the recommendations of the French Endocrine Society [46], a diagnosis of PAI in a patient under treatment with ICI should be considered in the following cases: (i) acute presentation of indicative symptoms or signs as described above, (ii) hyponatremia-related alteration in general status, (iii) isolated electrolyte imbalance (hyponatremia or hyperkalemia). A diagnosis of PAI involves evaluating cortisol levels in the morning, with a low baseline cortisol level (usually below 100 nmol/L or 5 µg/dL) and an ACTH level above two times the upper reference limit being indicative of PAI [45]. In cases where baseline cortisol levels are between 5 and 18 µg/dL, a Synacthen (corticotropin) stimulation test may be performed [46]. A peak cortisol level below 500 nmol/L (18 µg/dL) at 30 or 60 min during the test is considered to be indicative of PAI. In addition to high ACTH levels, PAI is characterized by increased renin activity or concentration and decreased aldosterone concentration, as well as frequently occurring hyperkalemia, hyponatremia, and hypoglycemia [45].

The recent ESE’s guidelines [4] suggest the assessment of serum 21-OHAbs and a non-urgent adrenal CT scan to evaluate adrenal inflammation or atrophy and to rule out other potential secondary causes [4]. However, due to the rarity of PAI, there is not enough data to recommend systematic screening for PAI before or during immunotherapy, unless clinical signs suggest it [46].

#### 3.1.6. Management

Based on the ESE guidelines [45], patients with suspected adrenal crisis should be treated with an immediate parenteral injection of 100 mg (50 mg/m^2^ for children) hydrocortisone, followed by appropriate fluid resuscitation and 200 mg (50–100 mg/m^2^ for children) of hydrocortisone/24 h (via continuous IV therapy or 6 hourly injection) prior to the availability of the results of diagnostic tests. Once a patient’s condition is stable and the diagnosis has been confirmed, parenteral glucocorticoid therapy should be tapered over 3–4 days and an oral maintenance dose can be instituted. In case of acute PAI, ICI can be discontinued, but should be reintroduced when the steroid replacement has normalized blood electrolytes and the patient’s symptoms have resolved [4,46].

High-dose glucocorticoids are not recommended for the treatment of chronic PAI since there is no established efficacy and, additionally, they may induce SAI [4]. Thus, it is recommended to initiate hydrocortisone at a dose of 15–25 mg or cortisone acetate 20–30 mg divided into two or three daily doses or a dual-release hydrocortisone tablet 20 mg once daily [47]. Prednisolone at 3–4 mg daily is also an alternative option, while dexamethasone has no place in replacement therapy. Patients with PAI generally receive mineralocorticoid replacement comprised of fludrocortisone 0.05–0.15 mg/day [45]. Patients should be informed regarding the management of PAI and the essential need to increase the dose of glucocorticoid during minor or major stress to prevent adrenal crises. Regardless of ICI discontinuation, PAI remains permanent in most cases and glucocorticoid or mineralocorticoid replacement should not be interrupted abruptly without testing of adrenal function with dynamic tests (standard 250 μg Synacthen test) [4]. 

### 3.2. Diabetes Insipidus (DI) 

#### 3.2.1. Background

Central DI (CDI), or as it is recently renamed “central arginine vasopressin (AVP) deficiency” [48], is the most common form of DI and is generally the result of hypothalamic-neurohypophysial dysfunction leading to inadequate AVP secretion from the posterior pituitary or inadequate production from the hypothalamus Although hypophysitis is a common ICI complication, involvement of the posterior pituitary with CDI is overall rare, described in 2% and in 3% of anti-CTLA-4- and anti-PD-1-induced hypophysitis cases, respectively [49]. 

#### 3.2.2. Case Studies 

According to the WHO global database of individual case safety reports, between January 2011 and March 2019 [50], 16 CDI cases (0.26%) out of a total 6089 endocrine irAEs were reported. In addition, Zhai et al. [51] reviewed 24 cases (0.4%) of immune-related CDI in a total of 6260 endocrine irAEs reported to the FDA Adverse Event Reporting System (FAERS) database from 2014 to 2019.

ICI treatment has been reported to dysregulate the posterior pituitary–hypothalamic axis in 15 individual cases (12 males and 3 females) included in Table 2 [52,53,54,55,56,57,58,59,60,61,62,63,64,65]. Most patients developed CDI after a period ranging from 28 to 270 days following treatment, with the exception of one patient who developed CDI immediately after receiving sintilimab, a PD-1 inhibitor [61]. Five patients out of these 15 cases, had received monotherapy with CTLA-4 inhibitor (ipilimumab); *n* = 5 patients with PD-1 inhibitors (*n* = 4 with nivolumab and *n* = 1 with sintilimab); *n* = 2 patients with PD-L1 inhibitor (avelumab, atezolizumab); and *n* = 3 patients had received combination treatment with nivolumab and ipilimumab (*n* = 2) or tremelimumab and durvalumab (*n* = 1). At least five patients [52,57,59,63,64] had received ICIs as first-line systematic therapy for advanced disease. Notably, in one case, whole brain radiotherapy had preceded the ICI therapy, and thus, the mechanism of panhypopituitarism was not clear [53].

In eight patients, CDI developed in the context of panhypophysitis [53,54,55,56,58,59,62,64], while in a further five patients [52,57,60,61,63] CDI developed in the context of isolated damage of the posterior pituitary. Although in one case CDI was accompanied with other anterior dysfunctions, it was considered to be the result of hypothalamitis based on clinical symptoms (severe sleep apnea and temperature dysregulation) and brain imaging (infiltrating, heterogeneously enhancing solitary lesion in the hypothalamus) [58]. It is important to note that the diagnoses of DI were based on the presence of polyuria/polydipsia symptoms, plasma or urine osmolality levels, and/or electrolytical disturbances. In seven cases [52,53,60,64,65], the diagnoses of CDI were well established based on water deprivation tests and/or copeptin levels. In the remaining cases [54,55,57,58,59,61,62,63], diagnoses of CDI were not validated by the recommended algorithm but were based on more “indirect” parameters such as relevant clinical symptoms related with concomitant anterior hypophysitis and/or indicative MRI finding (absent bright spot or hypothalamic mass) or response to desmopressin (improvement of the symptoms and normalization of electrolyte disturbances).

#### 3.2.3. Pathophysiology

The spectrum of ICI-induced hypothalamus–pituitary autoimmunity includes the anterior hypophysitis, infundibulo-neurohypophysis, panhypophysitis, and hypothalamitis, among which the anterior hypophysitis is the most common entity [66]. The higher incidence of anterior pituitary deficiency as compared with the rare manifestation of posterior pituitary dysfunction in ICI-induced hypophysitis may be attributed to the rich vascularity of the anterior pituitary gland with higher exposure to systemic therapy and its associated toxicities [57]. 

In the few reported ICI-induced CDI cases, the pathophysiological mechanism remained to be unclear, mainly because of the lack of autopsies and subsequent absence of histopathological analyses of patients’ impaired pituitary glands. The histopathological analysis of a single case of a patient with clinical signs of anterior hypophysitis showed that the pituitary gland presented type II and IV hypersensitivity reactions. Interestingly, strong CTLA-4 expression in the pituitary gland was also observed [67]. The posterior pituitary appeared to be normal, consistent with the absence of CDI in this patient. A recent report revealed the expression of PD-L1 receptor on hypothalamic cells which may explain the most frequent hypothalamic toxicity during anti-PD-L1 treatment [68]. Interestingly, all cases with posterior hypophysitis or hypothalamitis had been treated only with anti-PD1/PD-L1 agents or regimens [52,57,58,60,61,63].

#### 3.2.4. Clinical Presentation

Patients with CDI typically present with polyuria, nocturia, and polydipsia due to the initial elevation in serum sodium and osmolality [69]. There may also be symptoms of coexisting anterior pituitary dysfunction or hypothalamic dysfunction [58].

#### 3.2.5. Diagnosis

Hypotonic polyuria (urine output more than 50 mL/kg/24 h and urinary osmolality less than 300 mOsm/kg) associated with high plasma sodium concentration indicates a highly suspected diagnosis of DI. The differential diagnosis between CDI, nephrogenic DI, and primary polydipsia requires dynamic tests such as a water deprivation test. However, a water deprivation test often requires long periods of observation, and has considerable diagnostic limitations [69]. Recently, copeptin (C-terminal peptide of pro-AVP) level, either baseline or after stimulation (with hypertonic saline infusion or with L-arginine stimulation), has proven to be a promising biomarker for polyuria-polydipsia syndrome. Copeptin is co-secreted with AVP and is a surrogate of its secretion as it is a more stable compound. A baseline copeptin level >21.4 pmol/L has been found to be diagnostic of NDI, while an osmotically stimulated copeptin level of <4.9 pmol/L has been found to be diagnostic of CDI [69]. When a patient is treated with an ICI, distinct endocrine complications may mask the presence of CDI. In particular, aldosterone deficiency causes urinary sodium loss and cortisol deficiency impairs free water excretion. It is essential that these conditions be corrected before testing for AVP deficiency [4]. On an unenhanced T1-weighted MRI, CDI generally manifests as a pituitary ”bright spot” absence with or without enlargement (2–3 mm) of the pituitary stalk; however, this finding alone is not necessarily sufficient to support CDI diagnosis. The posterior pituitary bright spot is a manifestation of stored AVP, although it is “normally” missing in 52–100% of the general population [69]. At last, a diagnosis of ICI-induced CDI is established once other more frequent causes of CDI (e.g., traumatic injury, sellar/suprasellar lesions, vascular disorders, infections, or use of other medications such as glucocorticoids and opiates) have been excluded. 

#### 3.2.6. Management 

Desmopressin is the current standard of care for patients with ICI-related CDI and should be administered while carefully monitoring the sodium and fluid balance over 24 h [4].

### 3.3. Hypoparathyroidism

#### 3.3.1. Background

Hypoparathyroidism is most commonly the result of inadvertent removal or irreversible damage of parathyroid glands, in a clinical scenario of anterior neck surgery. Autoimmune hypoparathyroidism is the most frequent form of non-iatrogenic hypoparathyroidism in adults and it can either be isolated or part of autoimmune polyendocrine syndrome type-1 (APS-1) [70]. However, it is classified as an orphan disease by the European Commission (ORPHA:36913). Although rare, autoimmune hypoparathyroidism has been recorded to be manifested following the use of ICIs for cancer treatment. 

#### 3.3.2. Case Studies

There were two pharmacovigilance retrospective studies that reported ICI-induced hypoparathyroidism. Data from 2014 to 2019 in the FAERS database [51] and data from VigiBase between January 2011 and March 2019 [50] revealed 18 ICI-induced hypoparathyroidism cases out of 6260 cases (0.28%) of endocrine irAEs and 11 ICI-induced hypoparathyroidism cases out of 6089 cases (0.18%) of endocrine irAEs, respectively.

The analytical data from nine cases of ICI-related hypoparathyroidism are also summarized in Table 3 [71,72,73,74,75,76,77,78,79]. Patients’ ages ranged between 53 to 76 years. Four patients were treated with anti-PD-1 monotherapy, one patient was treated with anti-CTLA-4 monotherapy, and four patients were treated with a combination of ipilimumab and nivolumab. In at least three of these patients [71,73,78], ICIs were used as first-line monotherapy for advanced cancer stage. The time from ICI initiation to the onset of hypoparathyroidism ranged between 1 month and 1.5 years. In terms of prognosis, seven of nine (77.8%) patients presented with acute severe hypocalcemia (Grade III/IV) requiring hospitalization. The diagnosis was based on hypocalcemia along with low PTH levels in all patients. Various autoantibody measurements were performed in six out of the nine patients. Ca2+-sensing receptor (CaSR) antibodies were analyzed in five patients and were found to be positive in four of the patients [71,74,76,77] and nonspecific in the last patient [75]. In addition to CaSR antibodies, *NACHT* leucine-rich repeat protein 5 (NALP5) antibodies and antibodies against cytokines were also measured in two of the patients [71,74] and they were found to be negative. In the last case, anti-parathyroid antibodies were measured and were also found to be negative [72]. Hypoparathyroidism was not described as a component of APS-1 in any of the above cases. According to the available follow-up data, all cases required continuous calcium and active vitamin D supplementation.

#### 3.3.3. Pathophysiology

The mechanism of ICI-related hypoparathyroidism remains unclear. Even though the expression of PD-1/PD-L1 or CTLA-4 is unknown in normal parathyroid tissue, PD-1 expression was demonstrated in 30% of 28 parathyroid carcinomas and in 49% of 63 parathyroid adenomas [80]. The underlying mechanism of ICI-related hypoparathyroidism likely involves the activation of CaSR antibodies that inhibit PTH secretion [71,74,76,77] or/and increased T-cell activity against parathyroid tissue [75].

#### 3.3.4. Clinical Presentation

Hypoparathyroidism may be associated with a spectrum of clinical manifestations, ranging from mild (perioral numbness, paresthesias of the hands and feet, muscle cramps) to severe (carpopedal spasm, laryngospasm, and focal or generalized seizures) symptoms of neuromuscular irritability. The duration, severity, and rate of development of hypocalcemia determine the clinical presentation [70]. 

#### 3.3.5. Diagnosis 

Persistent hypocalcemia (total serum calcium concentration <2.1 mmol/L) with a low or inappropriately normal parathyroid hormone (PTH) level and hyperphosphatemia is, in the absence of hypomagnesemia, diagnostic of hypoparathyroidism [81]. A possible autoimmune etiology of the patient’s hypoparathyroidism should be suspected if no history of past radiation to the neck or prior neck surgery or familial hypocalcemic disorders are present. 

As nonsurgical hypoparathyroidism is extremely rare, APS-I should be considered in every patient with hypoparathyroidism [82]. The testing for autoantibodies to type 1 interferons (detected in 95% of patients with APS-1) is a cost-effective tool for first-line screening [83]. The disease is the most common endocrine component of APS-1, manifested in more than 80% of adult patients with APS1. In APS-1 patients, NALP5 is the main immunological target in the parathyroid cells [82]. Measurement of CaSR antibodies may also be helpful in the diagnosis of autoimmune hypoparathyroidism [84]. However, in general, identification of autoimmune hypoparathyroidism is a diagnostic challenge due to the lack of specific immunological markers [85]. Consequently, a diagnosis of ICI-associated hypoparathyroidism is based on clinical criteria.

#### 3.3.6. Management

According to the recent ESE guidelines [4], immune-related hypoparathyroidism is treated similarly to hypoparathyroidism due to other causes. The major goal is to correct symptomatic hypocalcemia and to avoid short- and long-term complications, especially cardiological complications such as prolongation of the QT interval. Oral calcium and vitamin D supplementation is the standard treatment used, while recombinant PTH and high dose use of glucocorticoids are not recommended. In the case of acute severe symptoms, intravenous calcium gluconate along with oral calcium supplements and active vitamin D are required [70].

### 3.4. Lipodystrophy

#### 3.4.1. Background 

Lipodystrophy syndromes are a heterogeneous group of diseases, characterized by selective absence of adipose tissue. There are four major subtypes according to the pattern of adipose tissue loss and the manner of acquisition: congenital generalized lipodystrophy (CGL), acquired generalized lipodystrophy (AGL), familiar partial lipodystrophy (FPLD), and acquired partial lipodystrophy (APL) [86]. Recently, AGL and APL have been reported during ICI therapy. 

#### 3.4.2. Case Studies 

Seven case reports were recognized that described immune-related lipodystrophy (Table 4) [87,88,89,90,91,92,93]. Six patients were females, and one patient was male with ages between 34 and 67 years. All patients were on anti-PD-1 agents. In five patients, ICIs were used as the first-line treatment for advanced disease [87,88,89,90,91]. The onset of lipodystrophy symptoms ranged from 6 weeks to approximately 1.5 years from ICI initiation.

All but one patient developed AGL. The majority of patients also presented metabolic abnormalities. Three of the patients developed new-onset DM [87,91,93], while two patients experienced deterioration of their preexisting DM [88,92]. The same patients also presented excessive hypertriglyceridemia [87,88,91,92,93], while three patients had also developed hepatic steatosis [87,91,93]. Leptin and adiponectin levels were measured in five patients. All [87,88,91,92] but one patient [89] had low serum levels of adipocytokines. Regarding leptin levels, hypo- and hyperleptinemia have both been described, making leptin measurement unreliable as a disease marker.

Treatment was focused on managing the metabolic abnormalities. Glucocorticoid treatment was suggested in three cases of AGL. In the literature, there was no report of a patient who had been treated with metreleptin (recombinant human methionyl leptin).

#### 3.4.3. Pathophysiology

Although the mechanism of ICI-associated lipodystrophy has not been studied sufficiently, there is evidence of an immune-mediated destruction of adipose tissue. Inflammation and infiltration of fat tissue with CD3^+^ and/or cytotoxic CD8^+^ lymphocytes identified in histopathological analyses of subcutaneous fat of ICI-treated patients, strongly suggest an immune-mediated reaction against adipose tissue [87,88,89,91,92,93].

#### 3.4.4. Clinical Presentation

The main feature of lipodystrophy is the selective loss of subcutaneous adipose tissue leading to local body deformation. Although patients with AGL comorbidities such as insulin-resistant DM, dyslipidemia, non-alcoholic fatty liver disease, renal or reproductive dysfunction, as well as heart disease are frequent and potentially severe, they are rarely present in patients with APL [94]. 

#### 3.4.5. Diagnosis

The diagnosis of lipodystrophy was usually made clinically based on history, physical examination, and a metabolic profile indicating non-responsive to therapy. In addition, a substantial subset of patients with lipodystrophy exhibited low leptin level. However, there was no defined serum leptin level that established or ruled out the diagnosis [94]. The identification of underlying etiology may be challenging in the context of cancer immunotherapy, given the lack of specific testing and wide differential diagnosis. However, an ICI-related mechanism should be suspected if further evaluation (physical features, family history, disease onset, past history and medical treatment, clinical and serological evaluation for autoimmune diseases, and genetic testing) rules out alternative causes.

#### 3.4.6. Management

For the generalized form, treatment of the accompanying metabolic symptoms is of primary concern. Currently, metreleptin is the only drug approved specifically for lipodystrophy [94]. Further studies should evaluate the role of leptin replacement therapy in ICI-associated AGL.

### 3.5. Osteoporosis

#### 3.5.1. Background

Osteoporosis is developed under multiple factors, including genetic factors as well as a range of other acquired and modifiable risk factors. Recently, there has been increasing interest to study the immune involvement in its pathogenesis [95]. Although current guidelines provide no evidence of direct or indirect effect of ICI therapy on bone metabolism, some published data have proposed that immune activation by ICIs may adversely impact bone remodeling.

#### 3.5.2. Case studies

Three published case series described skeletal-related events (SRE) in patients undergoing ICI therapy for different types of cancer (Table 5). One of the case studies [96] identified three patients without a prior diagnosis of osteoporosis presenting with new fractures during the course of anti-PD-1 therapy. Their ages ranged from 52 to 79 years and none of them had apparent pre-existing bone loss risk factors including focal bone radiation, family history of osteoporosis, tobacco or alcohol abuse, renal disease, or prolonged corticosteroid use. The study reported three more patients who developed new destructive or resorptive bony lesions that were not consistent with metastases during ICI treatment. At the biochemical level, elevated or high-normal bone turnover markers (C-telopeptides/bone-specific alkaline phosphatase) were reported in five out of these six patients, while inflammatory markers (C-reactive protein/erythrocyte sedimentation rate) were elevated in all patients.

The second case series by [97] reported four patients with no previous history of osteopenia or osteoporosis who developed osteoporotic fractures while being treated systemically with ICIs. Patients were from 62 to 70 years old at the time of development of SRE, and mainly females (3 patients of 4). Pre-existing risk factors for osteoporosis included smoking in three patients (<40 pack/years), mild renal failure in two patients, and long-term use of proton pump inhibitors (PPIs) in all four patients.

Moreover, an analysis of the worldwide pharmacovigilance database FAERS from 2014 to 2020 reported a statistically significant odds ratio (OR) for pathological fracture (*n* = 46, OR = 3.17, 95% CI 2.37–4.24), spinal compression fracture (*n* = 42, OR = 2.51, 95% CI 1.91–3.40), and femoral neck fracture (*n* = 26, OR = 2.38, 95% CI 1.62–3.50) in patients treated with ICI (specially with PD-1 inhibitors) compared to patients who received any other drug reported in FAERS [97]. Potential confounding clinical conditions or suspicious drugs were reported in limited cases. 

The last study by [98] evaluated the changes in plasma levels of bone turnover markers (collagen C-terminal telopeptide (CTX-1) and *N*-terminal propeptide of type I procollagen (PINP)) in 44 patients (median age = 70 years) affected by non-small cell lung cancer (*n* = 36) or renal cell cancer (*n* = 8) after 3 months of anti-PD-1 monotherapy. The patients had neither prior history of osteoporosis nor preexisting risk factors. CTX-1 levels were significantly increased, while PINP levels showed a trend of decreasing compared to baseline levels before ICI initiation. Interestingly, 4 of these 44 patients (9%) developed new diagnosed lumbar fractures during the 3-month follow-up. Notably, in at least four patients who developed skeletal effects, ICIs were used as the first-line treatment for advanced disease [96]. 

#### 3.5.3. Pathophysiology

The immune system is considered to play an integral role in the pathogenesis of osteoporosis [95]. Activated immune cells either directly or indirectly through the secretion of various cytokines and factors regulate bone remodeling. Immune activation induced by ICIs may impact bone metabolism, leading to bone resorption stimulation. In the setting of ICIs, activated T cells that secrete cytokines (TNF-α, IL-1, -4, -6, IL-17, and IFN-γ) have been associated not only with tumor cell destruction [99] but also with osteoclast formation and skeletal degradation [100]. 

#### 3.5.4. Clinical Presentation

Osteoporosis has no clinical manifestations until there is a fracture. Fractures may cause acute pain and loss of function but may also occur without symptomatology [101]. 

#### 3.5.5. Diagnosis 

The operational definition of osteoporosis is based on a T-score for bone mineral density (BMD) assessed by dual-energy X-ray absorptiometry (DXA) at the femoral neck or spine. Diagnostic work-up will depend on the severity of the disease, the age at presentation. and the presence or absence of vertebral fractures [101]. A correlation among clinical history, time course, and the absence of risk factors or underlying diseases, remains the major key to differentiate a case of ICI-related osteoporosis from the other causes. 

#### 3.5.6. Management 

The treatment of osteoporosis consists of lifestyle measures and pharmacologic therapy. Lifestyle measures include adequate calcium and vitamin D, exercise, smoking cessation, counseling on fall prevention, and moderation of alcohol use. Anti-resorptive and anabolic agents are alternative effective treatment options for certain subsets of patients [101]. 

### 3.6. Hypergonadotropic Hypogonadism

ICIs may evoke disorders in the reproductive activity either inducing secondary hypogonadism in the context of hypophysitis with follicle-stimulating hormone (FSH) and luteinizing hormone (LH) insufficiency (secondary hypogonadism) or rarely targeting directly the gonads (testes and ovaries) causing impaired oogenesis/spermatogenesis and consequently fertility compromise [102]. The incidence of secondary hypogonadism increases to 85% of the ICI-treated cases with hypophysitis, while primary hypogonadism has been described only in anecdotal cases (Table 6).

Primary hypogonadism, affecting sperm and/or testosterone production, has been described in 13 male patients treated with ICIs. However, it was unclear whether these cases represented true irAEs. In at least nine patients, ICIs were used as a first-line systematic treatment for advanced disease [104,105,106,107]. However, in another three patients, there were also additional risk factors which could be incriminated for impaired spermatogenesis (radiotherapy to inguinal lymph nodes (*n* = 1), alcohol abuse (*n* = 1), prior history of orchitis (*n* = 1)), and thus, ICI treatment could not be identified as fully responsible for the hypogonadism [106]. Moreover, notably, a baseline (before any treatment) spermogram was available and was normal in only one case [106]. Testicular biopsies, when performed, revealed an inflammation infiltrate without any specific finding of a potential ICI-related pathogenetic mechanism. Notably, PD-L1 and PD-L2 are normally expressed along the female reproductive tract, with differential expression depending on menopausal status, serving as potential direct targets of ICI therapy [108]. However, to the best of our knowledge, to date, no case of ICI-related primary hypogonadism has been described in the female population.

### 3.7. Cushing Disease (CD)

Cushing disease (CD) is an extremely rare adverse event which was described thoroughly in a single case report in [109] in the context of destructive hypophysitis during immunotherapy with nivolumab and ipilimumab for the treatment of metastatic melanoma. The patient was a 53-year-old female who presented typical signs and symptoms of CD manifested within the 9th and the 11th week of the immunotherapy initiation. Biochemical testing confirmed an ACTH-dependent hypercortisolism. Hormonal pituitary assessment also showed concomitant gonadotroph and thyreotroph deficiency. Inferior petrosal sinus sampling determined the pituitary origin of the ACTH hypersecretion and a pituitary MRI showed enlargement of the pituitary gland. Four weeks after the diagnosis of CD, permanent SAI was developed, while no recurrence of CD was observed. According to a population-based analysis of 4489 patients with melanoma, ICI-treated patients had a greater risk to develop CD (HR = 11.8, 95% CI 1.4–97.2) compared to those who did not receive immunotherapy [41].

## 4. Future Aspects

It would be interesting and rather challenging to be able to predict the occurrence of endocrine irAEs in candidates for immunotherapy. Some data suggest that certain biochemical biomarkers measured at baseline before any ICI treatment could predict irAEs. For instance, thyroid-stimulating hormone levels or antithyroid antibodies, as well as cytokines (interleukins IL-2 and IL-1b) measured at baseline could predict thyroid dysfunction. Similarly, specific HLA alleles or anti-pituitary antibodies screening before ICI treatment could predict the occurrence of hypopituitarism and especially deficiency of ACTH [110].

## 5. Conclusions

A systemic registry of all endocrine irAEs could prove to be very important for the evaluation of their exact frequency. It is also mandatory before attributing an endocrine irAEs to ICI therapy to run a specific diagnostic pathway exploring the autoimmune background (antibodies, biopsy) following the current guidelines. Indeed, the diagnosis of rare and very rare irAEs remains to be doubtful, particularly in multi-treated cases in whom ICIs are the second- or third-line option, and thus, there is an accumulative toxicity from previous therapies. The diagnosis and the management of rare and very rare endocrine irAEs should both be guided by a multidisciplinary team on an individualized basis. Physicians and healthcare providers should be aware of the manifestation of these endocrine toxicities, despite their rarity, for their early diagnosis and treatment. As the use of ICIs expands, it is also important to develop registries with long-term follow-up to better monitor, record, and understand these rare immune related endocrinopathies.

## Figures and Tables

**Table 1:** Cases in the literature presenting ICI-related primary adrenal insufficiency (PAI).

Reference	Type of Study, (*n*)	Age (y)	Sex (M, Male and F, Female)	Malignancy	Drug	ICI Category	Previous Therapies	Laboratory Evaluation	Adrenal Imaging Findings after ICI Initiation (Method)	Grade of AE	Onset after ICI Initiation (Days)	Outcome of AE	Follow-Up (Days)
Abdallah et al., 2020 [20]	Case report (*n* = 1)	70	F	Pancreatic adenocarcinoma	Nivolumab	PD-1 Ab	ND	Normal sodium and potassium levels, low F and increased ACTH levels, 21-OH Abs (-)	Normal (ND)	IV	After 3rd dose	ND	90
Agrawal et al., 2019 [15]	Case report (*n* = 1)	59	M	SCLC	Ipilimumab + Nivolumab	CTLA-4+PD-1 Ab	Lung radiotherapy and chemotherapy	Low F and increased ACTH levels	ND	II	120	ND	ND
Akarca et al., 2017 [14]	Case report (*n* = 1)	52	M	NSCLC	Nivolumab	PD-1 Ab	ND	Hyponatremia and hyperkalemia, low F and increased ACTH levels	Normal (ND)	IV	14	ND	ND
Bacanovic et al., 2015 [30]	Case report (*n* = 1)	79	ND	ND	Ipilimumab	CTLA-4 Ab	ND	ND	Symmetrically and smoothly enlarged, hypermetabolic (FDG-PET/CT)	ND	ND	ND	ND
Bischoff et al., 2022 [23]	Case report (*n* = 1)	53	F	Melanoma	Pembrolizuma	PD-1 Ab	Surgery	Hyponatremia, hyperkalemia, low F and increased ACTH levels, ACA (+), 21-OH Abs (+)	Normal (FDG-PET/CT)	III-IV	168	ND	ND
Coskun et al., 2016 [32]	Case report (*n* = 1)	50	M	Lung adenocarcinoma	Nivolumab	PD-1 Ab	ND	Hyponatremia, hyperkalemia, low F and increased ACTH levels	Normal (Ultrasound)	III	10	ND	ND
Dasgupta et al., 2022 [28]	Case report-APS-2 (*n* = 1)	14	F	Hepatocellular carcinoma	Nivolumab	PD-1 Ab	Chemotherapy	Normal morning F and ACTH levels, 21-OH Abs (+) ^a^	ND	I	ND	ND	ND
Deligiorgi et al., 2020 [26]	Case report (*n* = 1)	42	M	Rectal adenocarcinoma	Nivolumab	PD-1 Ab	Surgery and chemotherapy	Hyponatremia, low F and increased ACTH levels, low PAC levels, 21-OH Abs (+)	Normal (CT)	III–IV	112	Recovery after 12 weeks	630
Figuerora-Perez et al., 2021 [29]	Case report (*n* = 1)	73	M	Renal cell carcinoma	Pembrolizumab	PD-1 Ab	Surgery and axitinib	Low F and increased ACTH levels, low PAC levels, high PRA, 21-OH Abs (-)	ND	II	ND	ND	ND
Gaballa et al., 2020 [34]	Case report (*n* = 1)	76	M	Melanoma	Ipilimumab	CTLA-4 Ab	None	Hyponatremia with normal potassium, low F and elevated ACTH levels, PAC levels undetectable, increased PRA levels	Normal (CT)	III	After 4 cycles	Recovery	16 cycles of nivolumab
Galliazzo et al., 2022 [38]	Case report (*n* = 1)	74	M	NSCLC	Nivolumab	PD-1 Ab	ND	Hyponatremia, low F and increased ACTH levels, low PAC levels, 21-OHAbs (-)	Normal (CT)	ND	ND	ND	ND
Gunjur et al., 2019 [16]	Case report- APS-2 (*n* = 1)	78	F	Melanoma	Pembrolizumab	PD-1 Ab	None	Hyponatremia with normal potassium, Pathological cosyntropin stimulation test (Synacthen), HLA-DRB1*04 genotype (DR4 serotype)	Normal (FDG-PET/CT)	III–IV	63	Persistence	365
Hanna et al., 2018 [37]	Case report (*n* = 1)	70	M	Lung adenocarcinoma	Pembrolizumab	PD-1 Ab	None	Pathological cosyntropin stimulation test (Synacthen)	ND	III–IV	ND	ND	ND
Harsch et al., 2020 [21]	Case report (*n* = 1)	62	F	Melanoma	Pembrolizumab	PD-1 Ab	None	Hyponatremia with normal potassium, low F and increased ACTH levels	Inconspicuous adrenal glands (CT)	III–IV	365	ND	ND
Hescot et al., 2018 [39]	Case report (*n* = 1)	33	F	Cervical squamous cell cancer	Pembrolizumab	PD-1 Ab	ND	Hyponatremia with normal potassium, low F and increased ACTH levels, 21-OH Abs (+)	Adrenal hypoplasia (CT)	III–IV	147	Recurrence	365
Hobbs et al., 2020 [33]	Case report (*n* = 1)	58	M	ND	Ipilimumab + Nivolumab	CTLA-4 Ab+ PD-1 Ab	ND	Hyponatremia with hyperkalemia, low F and increased ACTH levels	ND	III	After 4 cycles	ND	ND
Iqbal et al., 2019 [18]	Case report (*n* = 1)	65	F	NCSLC	Nivolumab	PD-1 Ab	ND	Hyponatremia with hyperkalemia, low F and increased ACTH levels, low PAC levels with increased PRA	Normal (CT)	III	ND	Persistence	ND
Kagoshima et al., 2019 [19]	Case report (*n* = 1)	57	F	Tongue squamous cell carcinoma	Nivolumab	PD-1 Ab	Surgery, radiotherapy and chemotherapy	Low F and normal ACTH levels, CRH test in favor of PAI	ND	II	ND	ND	ND
Knight et al., 2021 [31]	Prospective study (*n* = 1)	59	M	Renal cell carcinoma	Ipilimumab + Nivolumab	CTLA-4 Ab+ PD-1 Ab	ND	Hyponatremia, low F and increased ACTH levels	ND	III–IV	ND	ND	ND
Kojadinovic et al., 2021 [36]	Case report (*n* = 1)	64	Μ	Colorectal cancer	Pembrolizumab (9 cycles), pembrolizumab+ Ipilimumab	PD-1 Ab +CTLA-4 Ab	Surgery and multiple cycles of chemotherapy	Hyponatremia and low F levels, pathological cosyntropin stimulation test (Synacthen)	ND	II	14 after initiation of dual therapy	Persistence	567
Lanzolla et al., 2019 [17]	Case report- APS-2 (*n* = 1)	50	M	Lung adenocarcinoma	Atezolizumab	PD-L1 Ab	Chemotherapy	Hyponatremia with normal potassium, low F and increased ACTH levels, low PAC levels with increased PRA, 21-OH Abs (+), HLA typing: DRB1*04 and DQB1*03 haplotypes	Normal (CT)	III	84	ND	ND
Min et al., 2013 [25]	Case report- mixed AI (PAI + SAI) (*n* = 1)	56	F	Melanoma	Ipilimumab	CTLA-4 Ab	ND	Low F and increased ACTH levels	Reversible bilateral enlargement (CT)	II	After 4 doses	ND	ND
Ozyurt et al., 2021 [22]	Case report (*n* = 1)	66	M	Renal cell carcinoma	Nivolumab	PD-1 Ab	Sunitinib	Hyponatremia with hyperkalemia, low F and increased ACTH levels, (under steroids)	ND	II	21	Persistence	60
Paepegaey et al., 2017 [13]	Case report-APS-2 (*n* = 1)	55	F	Melanoma	Pembrolizumab	PD-1 Ab	Surgery, chemotherapy and sorafenib	Hyponatremia with hyperkalemia, low F and increased ACTH levels, PAC undetectable with increased PRA levels, ACA (+), 21-OH Abs (+)	Atrophied adrenal glands (CT)	IV	258	ND	ND
Salinas et al., 2020 [27]	Case series (*n* = 3)	60, 65, 76	M(all)	Renal cell carcinoma(all)	Ipilimumab + nivolumab (*n* = 1) Nivolumab (*n* = 2)	-CTLA-4 Ab+ PD-1 Ab -PD-1 Ab (*n* = 2)	Cabozantinib (*n* = 1), none (*n* = 2)	Hyponatremia and pathological cosyntropin stimulation test (Synacthen) (*n* = 2) Pathological cosyntropin stimulation test (Synacthen) (*n* = 1)	Normal (*n* = 3) (CT)	IV IV III	140 183 150	ND	ND
Shariff et al., 2018 [35]	Case report (*n* = 1)	49	M	Melanoma	Ipilimumab + nivolumab	CTLA-4 Ab+ PD-1 Ab	Chemotherapy	Hyponatremia with hypokalemia, low F levels and increased ACTH levels, low PAC with increased PRA levels, 21-OH Abs (-)	Normal (ND)	III	74	Persistence	420
Trainer et al., 2016 [24]	Case study (*n* = 1)	43	F	Melanoma	Nivolumab	PD-1 Ab	Surgery	Hyponatremia, low F and increased ACTH levels, low PAC with increased PRA levels	Symmetrically and smoothly enlarged, increased FDG activity in both adrenal glands (FDG-PET/CT)	III	56	Persistence	365

Abbreviations: ICI, immune checkpoint inhibitors; ND, no data; CTLA-4, cytotoxic T-lymphocyte-associated protein 4; PD-1, programmed cell death protein 1; PD-L1, programmed cell death ligand 1; AE, adverse effect; NSCLC, non-small cell lung cancer; F, morning cortisol level; ACTH, adrenocorticotropic hormone, ACA: adrenal cortex antibody; 21-OH Abs: 21-hydroxylase antibodies; CRH: corticotropin-releasing hormone; PAC: plasma aldosterone concentration; PRA, plasma renin activity. ^a^ A case of developing overt PAI.

**Table 2:** Cases in the literature presenting with ICI-induced central diabetes insipidus (CDI).

Reference	Type of Study, (*n*)	Age (y)	Sex (M, Male and F, Female)	Malignancy	Drug	ICI Category	Previous Therapies	Dysfunction of Pituitary	Dysfunction of Hypothalamus	Onset after Initiation of ICI (Days)	Outcome of AE	Laboratory Evaluation	MRI Findings	Grade of AE	Follow-Up (Days)
Amereller et al., 2021 [65]	Retrospective study (*n* = 2)	ND	M (*n* = 1), F (*n* = 1)	ND	Ipilimumab	CTLA-4 Ab	ND	ND	ND	ND	ND	Serum and urine osmolarity, serum Na, water deprivation test (+)	ND	ND	ND
Angelousi et al., 2022 [64]	Case report (*n* = 1)	53	F	Melanoma	Nivolumab	PD-1 Ab	Multiple surgeries	Panhypopituitarism	ND	240	Persisted	Low urine osmolality, increased plasma osmolality, water deprivation test (+), low baseline copeptin levels	Absent bright spot	II	180
Barnabei et al., 2020 [59]	Case report (*n* = 1)	64	M	Melanoma	Ipilimumab	CTLA-4 Ab	Ocular proton beam radiotherapy	Panhypopituitarism	No	60	Transient (5 days)	Low urine osmolality, increased plasma osmolality, normal serum Na	Absent bright spot	I	1230
Brage et al., 2022 [53]	Case report (*n* = 1)	46	M	Adenocarcinoma of the lung	Nivolumab	PD-1 Ab	Whole brain radiotherapy, erlotinib osimertinib and chemotherapy	Panhypopituitarism	No	62	ND	Low urine osmolarity, water deprivation test (+)	ND	I	0
Brilli et al., 2020 [52]	Case report (*n* = 1)	68	M	Mesothelioma	Tremelimumab and durvalumab	CTLA-4 Ab + PD-L1 Ab	None	Isolated posterior pituitary	No	60	Persisted	Normal levels of serum sodium, plasma osmolality and urinary specific gravity test, water deprivation test (+)	Normal	ND	570
Deligiorgi et al., 2020 [60]	Case report (*n* = 1)	71	M	Adenocarcinoma of the lung	Nivolumab	PD-1 Ab	Surgery and chemotherapy	Isolated posterior pituitary	No	90	ND	Hypernatremia, high plasma osmolarity and hyposthenuria, undetectable serum AVP	Normal	IV	ND ^a^
Dillard et al., 2009 [55]	Case report (*n* = 1)	50	M	Adenocarcinoma of prostate	Ipilimumab	CTLA-4 Ab	ND	Panhypopituitarism	No	84	Transient (3 weeks)	ND	Normal	III	ND
Fosci et al., 2021 [62]	Case report (*n* = 1)	62	M	Hypopharynx cancer	Nivolumab	PD-1 Ab	Surgery and chemotherapy	Panhypopituitarism	No	35	50 days ^b^	Low urine osmolarity, high plasma osmolality, response to desmopressin	Enlarged stalk	I	50 ^b^
Grami et al., 2019 [54]	Case report (*n* = 1)	30	M	Acute myeloid leukemia	Ipilimumab + nivolumab	CTLA-4 Ab + PD-1 Ab	Chemotherapy and allogenic stem cell transplant	Panhypopituitarism	No	ND	ND	Low urine osmolarity, high serum Na, response to desmopressin	ND	III	ND
Gunawan et al., 2018 [57]	Case report (*n* = 1)	52	M	Melanoma	Ipilimumab+ nivolumab	CTLA-4Ab + PD-1 Ab	Small bowel resection	Isolated posterior pituitary	No	28	ND	High serum Na, response to desmopressin	ND	I	ND
Nallapanemi et al., 2014 [56]	Case report (*n* = 1)	62	M	Melanoma	Ipilimumab	CTLA-4 Ab	Vemurafenib+IL-2	Panhypopituitarism	No	121	5mo	Water deprivation test (+)	ND	II	180
Tshuma et al., 2018 [58]	Case report (*n* = 1)	74	F	Bladder cancer	Atezolizumab	PD-L1 Ab	Surgery + neoadjuvant chemotherapy	Panhypopituitarism	Yes	270	ΝD	High serum Na, low urinary Na	Hypothalamic mass	I	365
Yu et al., 2021 [61]	Case report (*n* = 1)	60	M	Hodgkin lymphoma	Sintilimab	PD-1Ab	Chemotherapy	Isolated posterior pituitary	No	Immediate	Transient (3 months)	High serum osmolality, high serum Na, low urine-specific gravity, response to desmopressin	Nodular signal	II	90
Zhao et al., 2017 [63]	Case report (*n* = 1)	73	M	Merkel cell carcinoma	Avelumab	PD-L1 Ab	None	Isolated posterior pituitary	No	112	Transients (6 weeks)	High serum os- molarity, low urine osmolarity, low urine specific gravity, high serum Na, response to desmopressin	Normal	I	240

Abbreviations: ICI, immune checkpoint inhibitors; MRI, magnetic resonance imaging; AE, adverse events; ND, no data; CTLA-4, cytotoxic T-lymphocyte-associated protein 4; PD-1, programmed cell death protein 1; PD-L1, programmed cell death ligand 1; IL-2, interleukin-2; Na, sodium levels; AVP, arginine *vasopressin*. ^a^ The patient deceased before initiation of treatment with desmopressin. ^b^ The patient deceased 50 days after desmopressin initiation.

**Table 3:** Cases in the literature presenting with ICI-induced hypoparathyroidism.

Reference	Type of Study	Age (y)	Sex (M, Male and F, Female)	Malignancy	Drug	ICI Category	Previous Therapies	Laboratory Evaluation	Grade of AE	Onset after Initiation of ICI (Days)	Outcome of AE	Follow-Up (Days)
Dadu et al., 2020 [71]	Case report (*n* = 1)	73	M	Melanoma	Ipilimumab + nivolumab	CTLA-4Ab + PD-1 Ab	None	Low Ca, P and Mg levels, undetectable PTH levels, low 25-OHD3 CaSR-Abs (+), NALP5 Abs (-), Cytokine Abs (-)	IV	28	Persisted	1185
Horinouchi et al., 2015 [79]	Phase I study (*n* = 1)	ND	ND	NSCLC	Ipilimumab	CTLA-4 Ab	Chemotherapy	ND	I/II	ND	ND	ND
Kawkgi et al., 2020 [72]	Case report (*n* = 1)	76	M	Melanoma	Ipilimumab + nivolumab	CTLA-4Ab + PD-1 Ab	ND	Low Ca, P and Mg levels, undetectable PTH levels, normal 25-OHD3 levels, anti-PTH Abs (-)	III	220(combination therapy), 160 (nivolumab monotherapy)	Persisted	77
Lupi et al., 2020 [77]	Case report (*n* = 1)	53	M	Lung adenocarcinoma	Pembrolizumab	PD-1 Ab	ND	Low Ca, normal P and Mg levels, inappropriate normal PTH levels, low25-OHD3 CaSR Abs (+)	IV	510	Persisted	270
Mahmood et al., 2020 [78]	Case report (*n* = 1)	71	M	Lung adenocarcinoma	Pembrolizumab	PD-1 Ab	Surgery and lung radiotherapy	Low Ca and PTH levels	II	45	Persisted	210
Piranavan et al., 2018 [74]	Case report (*n* = 1)	61	F	SCLC	Nivolumab	PD-1 Ab	Chemotherapy and radiotherapy	Low Ca, P and Mg levels, low PTH levels, normal 25-OHD3 levels, CaSR Abs (+), NALP5 Abs (-), Cytokine Abs (-)	IV	120	ND	ND
Trinh et al., 2019 [75]	Case report (*n* = 1)	53	ND	Melanoma	Ipilimumab + nivolumab	CTLA-4Ab + PD-1 Ab	ND	Low Ca and Mg levels, normal P, normal 25-OHD3 levels, low PTH levels, CaSR Abs insignificant titers	IV	28	Persisted	14
Umeguchi et al., 2018 [76]	Case report (*n* = 1)	64	M	NSCLC	Pembrolizumab	PD-1 Ab	Chemotherapy and lung radiotherapy	Low Ca levels, increased P levels, normal 1,25-(OH)_2_ D3, low PTH levels, CaSR Abs (+)	III	42	Persisted	ND
Win et al., 2017 [73]	Case report (*n* = 1)	73	M	Melanoma	Ipilimumab + nivolumab	CTLA-4Ab +PD-1 Ab	Local excision	Low Ca and Mg levels, low 25-OHD3 levels, low PTH levels	IV	45	Persisted	120

Abbreviations: ICI, immune checkpoint inhibitors; ND, no data; CTLA-4, cytotoxic T-lymphocyte-associated protein 4; PD-1, programmed cell death protein 1; Ab, antibody; AE, adverse effect; (N)SCLC, (non-)small-cell lung cancer; Ca, corrected calcium level; Mg, magnesium level; PTH, parathormone level; P, phosphate level; 25-OHD3, 25*-hydroxy*-cholecalciferol; 1,25-(OH)_2_ D3, *1,25-dihydroxycholecalciferol*; CaSR Abs, Ca-sensing receptor-activating autoantibodies; NALP5 Abs, NACHT leucine-rich-repeat protein 5 antibodies.

**Table 4:** Cases in the literature presenting ICI-related acquired lipodystrophy.

Reference	Type of Study, (*n*)	Age(y)	Sex (M, Male and F, Female)	Malignancy	Drug	ICI Category	Previous Therapies	Type of Lipodystrophy	Laboratory Evaluation	Onset after Initiation of ICI (Days)	Grade of AE	Treatment of AE	Outcome of AE
Bedrose et al., 2020 [88]	Case report (*n* = 1)	67	M	Melanoma	Pembrolizumab	PD-1 Ab	None	Generalized	Hyperglycemia and hypertriglyceridemia, normal values of liver enzymes, low leptin and adiponectin levels	42	II	Insulin + pioglitazone, Statin + fibrate+ omega-3 fatty acids	ND
Drexler et al., 2021 [90]	Case report (*n* = 1)	41	F	Melanoma	Nivolumab	PD-1 Ab	Inguinal lymph node dissection and local excision	Facial	Normal values of cholesterol, triglycerides, HbA1C	474	II	Steroids	Persistence
Eigentler et al., 2019 [93]	Case report (*n* = 1)	45	F	Melanoma	Nivolumab	PD-1 Ab	Local excision and IFN-a	Generalized	Hyperglycemia, hypertriglyceridemia, increased liver enzymes	360	II	Steroids, insulin and then, overlapping courses of empagliflozin, liraglutide and pioglitazone	Improvement in metabolic abnormalities
Gnanendran et al., 2020 [89]	Case report (*n* = 1)	34	F	Melanoma	Nivolumab	PD-1 Ab	Local excision	Generalized	Normal values of glucose, HbA1C, LDL high leptin levels	270	II	Steroids	Persistence
Haddad et al., 2019 [87]	Case report (*n* = 1)	47	F	Melanoma	Pembrolizumab	PD-1 Ab	None	Generalized	Prediabetes, low leptin and adiponectin levels, hypertriglyceridemia	60	III	Treatment for metabolic abnormalities	Persistence
Jehl et al., 2019 [91]	Case report (*n* = 1)	62	F	Melanoma	Nivolumab	PD-1 Ab	Local excision	Generalized	DM, hypertriglyceridemia, increased liver enzymes, low leptin and adiponectin levels	540	III	Insulin + metfromin	Improvement in metabolic abnormalities
Kruschewsky Falcao et al., 2019 [92]	Case report (*n* = 1)	57	F	Renal cell carcinoma	Nivolumab	PD-1 Ab	Local excision and sunitinib, pazobanib	Generalized	DM, hypertriglyceridemia, high LDL, low leptin levels	60	II	Steroids	Improvement in metabolic abnormalities

Abbreviations: ICI, immune checkpoint inhibitors; ND, no data; PD-1, programmed cell death protein 1; Ab, antibody; AE, adverse effect; IFN-a, interferon-a; DM, diabetes mellitus; LDL, low density lipoprotein; HbA1C, *hemoglobin* A1C.

**Table 5:** Cases in the literature presenting ICI-related skeletal events.

Reference	Type of Study	Age (y)	Sex (M, Male and F, Female)	Malignancy	Drug/ICI Category	Previous Therapies	Skeletal AE	Laboratory Evaluation	Grade of AE	Onset after ICI Initiation
Filippini et al., 2021 [97]	Case series (*n* = 4)	67.8 (mean age)	M (*n* = 1), F (*n* = 3)	Squamous cell carcinoma (*n* = 4)	Anti-PD-1 Ab (*n* = 2) Anti-PD-L1 Ab (*n* = 2)	ND	Dorsal vertebral (D12) fracture Calcaneal fracture Lumbar vertebral (L1) fracture Multiple vertebral (D7-L5) fractures	ND	II	From 2.5 to 15.5 months
Moseley et al., 2018 [96]	Case series (*n* = 6)	59.3 (mean age)	M (*n* = 5), F (*n* = 1)	Melanoma (*n* = 4), RCC (*n* = 1), lung adenocarcinoma (*n* = 1)	Pembrolizumab/PD-1 Ab (*n* = 2), Nivolumab/PD-1 Ab (*n* = 2), Nivolumab + ipilimumab/PD-1 Ab + CTLA-4 Ab (*n* = 2)	Wide local excision + axillary lymph node dissection + GM-CSF secreting allogeneic melanoma cell vaccine (*n* = 1) wide local excision + IFN-a+IL-2 (*n* = 1), none (*n* = 4)	1st patient: compression vertebral fractures (T6, T7, T10, T11, and T12); rib and pelvic fractures 2nd patient: compression vertebral fractures (T6–12, L1) 3rd patient: compression vertebral fracture (T11); lumbar osteomalacia 4th patient: Resorptive bone lesion of left shoulder 5th patient: Resorptive bone lesion of right wrist 6th patient: Resorptive bone lesion of right clavicle	Elevated or high normal CTX and/or bsALP levels (*n* = 5), Elevated CRP and/or ESR (*n* = 6)	II	1st patient: After 20 doses of pembrolizumab therapy 2nd patient: 8 cycles of nivolumab and IL-21 3rd patient: 10 months 4th patient: 8 months 5th patient: 18 months 6th patient: ND
Pantano et al., 2022 [98]	Case series (*n* = 4)	ND	ND	ND	ND	ND	Lumbar fractures	Relatively increased CTX-1 levels ^1^ Relatively decreased PINP levels ^1^	II	ND

Abbreviations: ICI, immune checkpoint inhibitors; RCC, renal cell cancer; NSCLC, non-small cell lung cancer; AE, adverse effects; GM-CSF, granulocyte-macrophage colony-stimulating factor; IFN-a, interferon a; IL-2, interleukin-2; CTX, C-telopeptides; bsALP, bone-specific alkaline phosphatase; 25OHD, 25-hydroxy vitamin D; CRP, C reactive protein; ESR, erythrocyte sedimentation rate; ND, no data,;CTX-1, collagen C-terminal telopeptide; PINP, N-terminal propeptide of type I procollagen. ^1^ Mean levels of all (44) patients studied after 3-month ICI use. CTX-1 levels significantly increased, while PINP levels decreased compared to baseline levels (before ICI initiation).

**Table 6:** Cases in the literature presenting ICI-induced primary (hypergonadotropic) hypogonadism.

Reference	Type of Study, (*n*)	Age (y)	Sex (M, Male and F, Female)	Malignancy	Drug	ICI Category	Previous Therapies	Clinical Presentation	Laboratory Evaluation	Testicular Biopsy	Onset after Initiation of ICI (Days)	Duration of AE (Days)	Follow up (Days)
Brunet-Possenti et al., 2016 [103]	Case report (*n* = 1)	54	M	Melanoma	Ipilimumab + nivolumab	CTLA-4 Ab+ PD-1 Ab	ND	Bilateral orchitis	Low testosterone with high LH levels	ND	14	7	28
Quach et al., 2019 [104]	Case report (*n* = 1)	69	M	Melanoma	Pembrolizumab	PD-1 Ab	Partial hepatectomy and RFA of liver lesions.	Bilateral epididymo-orchitis	ND	ND	60	35	80
Rabinowitz et al., 2021 [105]	Case report (*n* = 1)	30	M	Melanoma	Ipilimumab + nivolumab	CTLA-4 Ab+ PD-1 Ab	None	Infertility	Spermogram: Azoospermia, Hormone profile: Low testosterone levels with high FSH and normal LH levels	Sertoli-only pathology	730 (time of evaluation)	Persisted	180
Salzamann et al., 2021 [106]	Cross-sectional pilot study (*n* = 4)	44, 51, 30, 36	M	ND	Ipilimumab + nivolumab (*n* = 2), Pembrolizumab(*n* = 1), PD-L1 Ab (*n* = 1)	CTLA-4 Ab+ PD-1 Ab (*n* = 2), PD-1 Ab (*n* = 1), PD-L1 Ab (*n* = 1)	RT to inguinal lymph nodes (*n* = 1), Chemotherapy (4 years before) (*n* = 1), ND (*n* = 2)	None	Spermogram: Azoospermia (*n* = 3), Oligoasthenoteratozoospermia (*n* = 1) Hormone profile: normal (*n* = 2), high FSH levels (*n* = 2)	No signs of inflammation (*n* = 2), Inflammation infiltrate (*n* = 2)	>120	ND	ND
Scovell et al., 2020 [107]	Cohort study (*n* = 6)	ND	M	Melanoma	Ipilimumab/nivolumab/pembrolizumab	CTLA-4 Ab/ PD-1 Ab	None	ND	ND	Sertoli-only syndrome (*n* = 3), focal active spermatogenesis (*n* = 1), hypospermatogenesis (*n* = 2)	ND	ND	ND

Abbreviations: ICI, immune-check point inhibitors; ND, no data; CTLA-4, cytotoxic T-lymphocyte-associated protein 4; PD-1:,programmed cell death protein 1; PDL1, programmed cell death ligand 1; RFA, radiofrequency ablation; RT, radiotherapy; Ab, antibody; AE, adverse effect; Testo, testosterone level; LH, luteinizing hormone level; FSH, follicle stimulating hormone level; E2, estradiol level; UNL, upper normal limit
